# Climatic niche divergence and habitat suitability of eight alien invasive weeds in China under climate change

**DOI:** 10.1002/ece3.2684

**Published:** 2017-02-08

**Authors:** Ji‐Zhong Wan, Chun‐Jing Wang, Jing‐Fang Tan, Fei‐Hai Yu

**Affiliations:** ^1^ School of Nature ConservationBeijing Forestry UniversityBeijingChina

**Keywords:** climatically suitable habitat, ecological niche divergence, invasive plants, invasive range, Maxent, species distribution models

## Abstract

Testing climatic niche divergence and modeling habitat suitability under conditions of climate change are important for developing strategies to limit the introduction and expansion of alien invasive weeds (AIWs) and providing important ecological and evolutionary insights. We assessed climatic niches in both native and invasive ranges as well as habitat suitability under climate change for eight representative Chinese AIWs from the American continent. We used climatic variables associated with occurrence records and developed ecological niche models with Maxent. Interestingly, the climatic niches of all eight AIWs diverged significantly between the native and invasive ranges (the American continent and China). Furthermore, the AIWs showed larger climatic niche breadths in the invasive ranges than in the native ranges. Our results suggest that climatic niche shifts between native and invasive ranges occurred. Thus, the occurrence records of both native and invasive regions must be considered when modeling and predicting the spatial distributions of AIWs under current and future climate scenarios. Owing to high habitat suitability, AIWs were more likely to expand into regions of low latitude, and future climate change was predicted to result in a shift in the AIWs in Qinghai and Tibet (regions of higher altitude) as well as Heilongjiang, Jilin, Liaoning, Inner Mongolia, and Gansu (regions of higher latitude). Our results suggest that we need measures to prevent and control AIW expansion at the country‐wide level.

## 1 Introduction

Alien invasive weeds (AIWs) have a high potential to threaten plant diversity (Ansong & Pickering, 2015; Beaumont, Gallagher, Leishman, Hughes, & Downey, 2014; van Kleunen et al., 2015; Parker, 2012; Stratonovitch, Storkey, & Semenov, 2012). By altering ecosystem functioning, the uncontrolled expansion of AIWs may also cause severe crop yield losses (Chauhan, Singh, Kumar, & Johnson, 2011; Fahad et al., 2015; Parker, 2012). In addition, climate change may promote the expansion of AIWs in non‐native ranges (Beaumont et al., 2014). In response to recent climate change, AIWs are expected to track favorable climates with respect to growth and expand their ranges via dispersal and adaptation (Clements & Ditommaso, 2011; Sheppard, 2013; Stratonovitch et al., 2012). To explore expanded AIW ranges, many ecologists have used ecological niche modeling (ENM) to evaluate habitat suitability in invasive regions under climate change conditions (Beaumont et al., 2014; Collingham, Wadsworth, Huntley, & Hulme, 2000). Suitable habitats may provide appropriate conditions for alien plants to expand in non‐native ranges (Adhikari, Tiwary, & Barik, 2015; Beaumont et al., 2014; Costa, Medeiros, Azevedo, & Silva, 2013; Sheppard, 2013). Thus, ecologists use ENMs to assess the invasion risk of AIWs in invasive ranges (Adhikari et al., 2015; Sheppard, 2013). For example, Costa et al. (2013) used ENMs to predict the suitable habitat distributions of three invasive weeds in New Zealand under climate change conditions, and Beaumont et al. (2014) used ENMs to examine the expansion risk of an invasive weed in Australia under climate change conditions. Although ENMs are widely used to assess invasion by AIWs, it is necessary to consider the effects of climatic niche divergence on the habitat suitability of AIWs in potentially invasive regions.

Some studies have shown that climatic niches are conserved between the native and invasive regions (Petitpierre et al., 2012). This observation is the basis for the use of ENMs calibrated in the native range to assess the invasion risk of invasive plant species in the invasive range (Broennimann, Mráz, Petitpierre, Guisan, & Müller‐Schärer, 2014; Guisan, Petitpierre, Broennimann, Daehler, & Kueffer, 2014; Petitpierre et al., 2012). Climatic niche conservatism predicts that invasive plant species are likely to grow and survive in environments that strongly resemble their native ranges (Broennimann et al., 2012; Guisan et al., 2014; Petitpierre et al., 2012). Indeed, Petitpierre et al. (2012) have shown that climatic niche shifts are rare among 50 terrestrial plant invaders between Eurasia, North America, and Australia based on principal component analysis (PCA) and found that fewer than 15% of species have the shifts between their native and invasive climatic niche spaces. However, there is no consensus on this question given that in other studies, niche divergence has been shown to occur between native and invasive ranges along a gradient of temperature and precipitation for 22 plant species endemic or near endemic to Europe that have been naturalized in the USA (Early & Sax, 2014). Shifts in climatic niches are relatively frequent among European species invading North America (Dellinger et al., 2016). This may be because climate niche divergence can occur as a result of nonclimatic factors, such as seed dispersal, human activities, and sexual reproduction (Prentis et al. 2008; Donoghue & Edwards, 2014; Dellinger et al., 2016). For instance, if biotic factors, abiotic factors other than the climate, or dispersal barriers limit the distribution in either the native or the invasive range, then the breadth of climatic tolerances of the species is likely to be underestimated (Alexander & Edwards, 2010; Guisan et al., 2014; Sax et al., 2007). Such climatic niche divergence may result in uncertainties in the use of ENMs to predict the habitat suitability of AIWs.

AIWs are uncultivated and useless invasive plant species that seriously threaten the economy and ecosystem in the invasive ranges (Li, 1998; Shen, Gao, Eneji, & Chen, 2013; Xu & Qiang, 2011). Many AIWs have been introduced in China over the past 100 years, and they have caused great crop yield loss (Xu & Qiang, 2011; Zhang, 2003). At least two studies have examined the impact of climate change on the expansion of Chinese AIWs from the American continent (Qin, DiTommaso, Wu, & Huang, 2014; Xu, Peng, Feng, & Abdulsalih, 2014), but these studies did not test climatic niche divergence and thus may not accurately predict habitat suitability. To address such practical issues, we examined climatic niche divergence between native and invasive ranges and predicted the habitat suitability of eight representative AIWs in China under climatic change. We tested the following two hypotheses: (1) climatic niches of AIWs are divergent between native and invasive ranges (i.e., the American content and China), and (2) climate change can increase the habitat suitability of AIWs in China.

## 2 Materials and Methods

### 2.1 Study areas and species data

According to Xu and Qiang (2011), more than 100 AIWs have been introduced from the American continent and have expanded widely in China. In this study, we focused on AIWs for which the invasive range is mainland China and the native range is the American continent. Mainland China has a continental monsoon climate and considerable climatic variation (Domrös & Peng, 2012). Mountains, plateaus, and hills cover approximately 67% of the land area, while basins and plains cover the remaining 33% (Figure S1). The altitudes of western regions in China are generally higher than those in eastern regions (Figure S1). Data of administrative ranges for Beijing and Tianjin were combined with data from the Hebei Province as well as data for Shanghai in the Zhejiang Province, Chongqing in the Sichuan Province, and both Hong Kong and Macau in the Guangdong Province (Figure S1).

We selected eight AIWs that are widely distributed in China: *Amaranthus retroflexus*,* Amaranthus spinosus*,* Amaranthus viridis*,* Bidens pilosa*,* Conyza bonariensis*,* Conyza canadensis*,* Galinsoga parviflora*, and *Physalis angulata* (Li, 1998; Xu & Qiang, 2011). We obtained occurrence records for both the invasive and native ranges from the Global Biodiversity Information Facility (GBIF; www.gbif.org). For China, we also added some occurrences from the Chinese Virtual Herbarium (CVH; www.cvh.org.cn). The occurrence records from the native range (i.e., the American continent including North America, South America, and the Caribbean region), which we also used as ENM inputs, were obtained from GBIF. Species were selected for this study based on three criteria: (1) they have at least 40 occurrence records after duplicates were removed and the locality and taxonomy checked for each record for both native and invasive ranges to improve the ENM accuracy (Coudun & Gégout, 2006; Dellinger et al., 2016), (2) they have a wide distribution and long introduction history in China to avoid the assessment uncertainty of climatic niche shifts as a result of dispersal lags (Gallien, Douzet, Pratte, Zimmermann, & Thuiller, 2012), and (3) they are known to have a negative impact on a variety of endangered plant species and ecosystems (Table S1).

### 2.2 Climatic data

We used 19 bioclimatic variables at a 5.0‐arc‐minute spatial resolution (often referred to as “100 km^2^” resolution) to visualize the climatic niches of AIWs and assess these climatic niches using climatic niche divergence analysis and habitat suitability modeling (Hijmans, Cameron, Parra, Jones, & Jarvis, 2005). These climatic data were downloaded from the WorldClim database (http://www.worldclim.org/; detailed information in Table S2; Hijmans et al., 2005). A multicollinearity test was performed for 19 bioclimatic variables (Merow, Smith, & Silander, 2013). Variables with Pearson correlation coefficients of >0.8 or less than −0.8 were removed to eliminate multicollinearity in the ENM parameter estimates (Merow et al., 2013). The remaining eight bioclimatic variables were related to the distribution and physiological performance of the plants. Eight future bioclimatic variables, which match present‐day variables, were assessed using the pixel maps of three global climate models, that is, mohc_hadgem2, csiro_mk3_6_0, and cccma_canesm2 (for the period 2,070–2,099), downloaded from the International Centre for Tropical Agriculture (http://ccafs-climate.org). Representative concentration pathways (RCPs) of 4.5 (mean, 780 ppm; range, 595–1,005 by the year 2100; low‐concentration scenario) and 8.5 (mean, 1,685 ppm; range, 1,415–1,910 by 2100; high‐concentration scenario) were used to model future species distributions. RCP 8.5 assumes larger cumulative concentrations of carbon dioxide than RCP 4.5, resulting in a different pattern of climate change in response to varying concentrations of greenhouse gases and other pollutants (http://www.ipcc.ch/report/ar5/).

### 2.3 Habitat suitability modeling

#### 2.3.1 Modeling with Maxent

**Figure 1:**
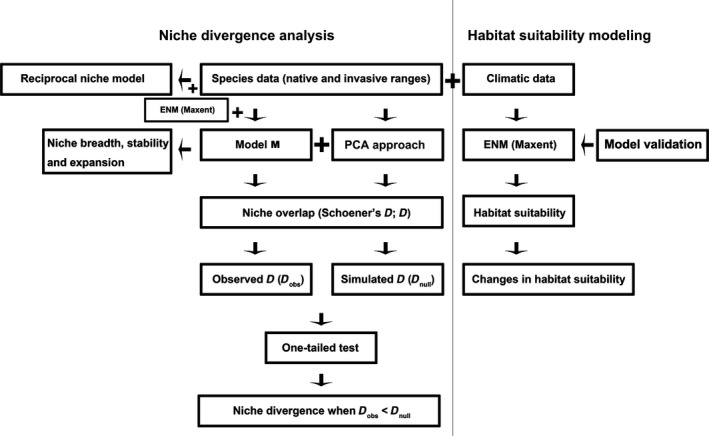
The flowchart for climatic niche divergence and habitat suitability analysis of the eight alien invasive weeds under climate change

Maxent (ver.3.3.3k; http://www.cs.princeton.edu/~schapire/maxent/) was used to model spatial distributions and produce habitat suitability maps of the AIWs for the present‐day scenarios based on bioclimatic variables and occurrence records (Merow et al., 2013). For the modeling, we accounted for occurrence records in both the invasive and native ranges because it improves the performance of ecological niche modeling (Merow et al., 2013; Mainali et al., 2015; Shabani & Kumar, 2015; Figure 1). In our study, three models were built using the occurrence records of native, invasive, and both native and invasive ranges, respectively. A fourfold cross‐validation approach was used to estimate the uncertainties in the response curves and occurrence predictions. The occurrence records were divided into four approximately equal random partitions. In turn, three of the partitions were used to train the model, while the fourth was used to generate the SDM estimate for its validation (each run used a different random sample points). Detailed information on the dataset of input occurrence records was shown for each AIW in Table S1. The maximum number of background points was set to 10,000. The convergence threshold was set to 0.0001. The regularization multiplier was fixed at two to generate a smooth and general response that could be modeled in a biologically realistic manner (Radosavljevic & Anderson, 2014). The maximum number of iterations was fixed to 500. All other parameters for Maxent were consistent with those of Phillips and Dudík (2008) and Elith et al. (2011).

#### 2.3.2 Validation

To assess the predictive capacity of the models, model predictions were compared with real observations (occurrences and pseudo‐absences) using the area under the curve (AUC) of a receiver‐operating characteristics plot (Fielding & Bell, 1997; Swets, 1988). This AUC measure allows us to test whether the obtained predictions differ significantly from a random prediction. As a rough guideline, models with AUC values below 0.7 were too poor to be considered in further analyses (Phillips & Dudík, 2008).

A binomial test (based on the training omission rate) was also used for model validation based only on present data. The training omission rate is the proportion of training occurrence records among the pixels of predicted absences (Phillips, Anderson, & Schapire, 2006; Phillips & Dudík, 2008). These are one‐sided tests (namely, one‐sided *p*‐values) for the null hypothesis that the Maxent modeling performs no better than random selection from the set of all models with similar proportional predicted areas (Phillips & Dudík, 2008; Phillips et al., 2006). A training omission rate of <17% was considered a good model performance (Phillips et al., 2006).

#### 2.3.3 Projection under future scenarios

First, the models were projected for the habitat suitability maps of AIWs under RCP 4.5 (the low‐concentration scenario) and RCP 8.5 (the high‐concentration scenario) based on the occurrence records of both invasive and native ranges and the bioclimatic variables in the three global climate models for the period 2,070–2,099. The current and future habitat suitability maps for each AIW were created using a binary distribution (i.e., a presence–absence pixel map) generated in ArcGIS 10.2 (Esri; Redlands, CA, USA) by reclassifying Maxent model pixels according to the 10th percentile training presence values (Callen & Miller, 2015). The habitat suitability maps for all eight AIWs were superimposed to produce the overall habitat suitability of AIWs under current‐, low‐, and high‐concentration scenarios.

Second, model projections were then used to complete the analyses of multivariate environmental similarity surface (MESS). Analyses in MESS were used to examine where novel climates arise in the future for each AIW (Callen & Miller, 2015). We averaged the MESS values across all eight AIW species to explore the overall occurrence of novel climates.

Third, we computed relative changes in the habitat suitability of each AIW individually between the current and the other two gas emission scenarios using the following formula: *A*_*t*_ = (*F*_*t*_ − *C*_*t*_)/*C*_*t*_, where *A*_*t*_ is the relative change in the habitat suitability of AIW *t* in China, *F*_*t*_ is the habitat suitability of AIW *t* in the future, and *C*_*t*_ is the current habitat suitability of AIW *t*. Then, we used Pearson correlation to evaluate the relationship between changes in the habitat suitability of the AIWs with respect to longitude, latitude, and altitude based on the occurrence records for each AIW to explore the biogeographical processes of AIWs under climate change conditions. Finally, we analyzed the overall habitat suitability of each AIW under each concentration scenario by calculating the proportion of presence pixels out of the total pixels in China and computed habitat suitability for the eight AIWs at the province scale using the following formula: *S*_*j*_ = ∑*Y*_*i*_ *,* (*i *=* *1, 2, 3…, *n*), where *S*_*j*_ is the current or future habitat suitability for the eight AIWs in province *j*,* n* is the total number of AIWs in province *j*, and *Y*_*i*_ is the percentage area of suitable habitat of AIW *i* in province *j* (Yu et al., 2014). We calculated relative changes in the habitat suitability of the AIWs under the current‐, low‐, and high‐concentration scenarios at the province scale based on the relationship *B*_*j*_ = (*F*_*j*_ − *C*_*j*_)/*C*_*j*_, where *B*_*j*_ is relative change in the habitat suitability for the eight AIWs in province *j*,* F*_*j*_ is the habitat suitability of the AIWs in province *j* in the future, and *C*_*j*_ is the habitat suitability of the AIWs in province *j* presently. We used linear regression to analyze the relationship between relative changes in habitat suitability for all eight AIWs with respect to longitude, latitude, and altitude based on the geographic center of the province.

### 2.4 Niche divergence analysis

We used three different and complementary methods to assess the overlap between invasive and native niches, that is, those of Broennimann et al. (2012), Warren, Glor, and Turelli (2008), and the reciprocal niche models (RNMs). Our approach is conceptualized in Figure 1.

#### 2.4.1 Overlap in climatic space

Based on the method developed by Broennimann et al. (2012), a PCA approach was used to test the differences in climatic niche between native and invasive ranges by quantifying niche overlap (Schoener's *D*; henceforth, *D*), niche stability, and expansion into the climatic niche space (Cornuault, Khimoun, Cuneo, & Besnard, 2015; Guisan et al., 2014). First, based on the observed occurrences for each species, a two‐dimensional climatic space was defined by the first two axes identified by the PCA. (Broennimann et al., 2012; Kumar et al., 2015). Second, this climatic space gridded with the first two principal components (PCs) was defined with 100 bins along each axis, thus partitioning the 2D climatic space into the gridded PCA climatic spaces (at a resolution of 100 × 100 cells), in which each cell corresponds to a unique combination of climate conditions (i.e., available environments; Cornuault et al., 2015; Dellinger et al., 2016). Here, a kernel function was used to smooth the climatic space defined in the gridded PCA climatic spaces based on the first two PCs (Petitpierre et al., 2012). Third, the observed *D* (*D*_obs_) was measured based on the occupancies in the climatic space defined by the gridded climatic spaces (Petitpierre et al., 2012). Niche stability is the proportion of the invasive distribution that overlaps with the native distribution (Petitpierre et al., 2012). Niche expansion is the proportion of the invasive distribution located in conditions that differ from those of the native distribution (or 1—stability; Petitpierre et al., 2012). Expansion measured in this way characterizes true climatic niche shifts (Petitpierre et al., 2012). Randomizations of the data were repeated 100 times, generating null distributions of simulated *D* (*D*_null_) values for comparison with *D*_obs_. For the equivalence test, the null hypothesis was rejected if *D*_obs_ was outside of the 95% confidence limits of *D*_null_. Niche divergence was inferred when *D*_obs_ < *D*_null_ (Callen & Miller, 2015). The “ecospat” package in R was used to conduct this analysis (https://www.r-project.org/).

Based on the method proposed by Warren et al. (2008), we used Maxent modeling to project the habitat suitability maps using the occurrence records from the invasive range, native range, and combined invasive and native ranges. Maxent modeling was performed using default values for the habitat suitability maps (Warren et al., 2008). Niche breadth, a metric with values ranging from 0 to 1, represents the correlation between the environmental range and the habitat suitability of a plant species. In other words, niche breadth is an indicator of the expansion potential of AIWs (Warren et al., 2008). ENMTools 1.4.4 was used to compute the climatic niche breadth using the habitat suitability maps based on invasive ranges, native ranges, and both invasive and native ranges (Warren, Glor, & Turelli, 2010; Warren & Seifert, 2011). Paired *t*‐tests were used to evaluate the differences in the climatic niche breadth among the invasive ranges, native ranges, and combined invasive and native ranges. The similarity between climatic niches in the native and invasive ranges was examined based on niche overlap (i.e., *D*) using ENMTools 1.4.4 (Warren et al., 2010). *D* ranges from 0 (no similarity) to 1 (completely overlapping; Warren et al., 2008). A null model was created to test climatic niche divergence between the native and invasive ranges. The background randomization test in ENMTools 1.4.4 was then used to test the null model that the observed niches and the AIW background environment were divergent using Maxent modeling (McCormack, Zellmer, & Knowles, 2010; Warren et al., 2008). First, the observed niche overlap of each AIW between native and invasive distributions (*D*_obs_) was determined using the Maxent modeling of the occurrence records of native and invasive regions. Second, the background randomization test was used to generate 100 pseudoreplicate datasets for Maxent modeling based on the combined native and invasive ranges (Hill, Hoffmann, Macfadyen, Umina, & Elith, 2012). Each pseudoreplicate dataset included an equal number of background points within native and invasive ranges to compute the simulated niche similarity from null distributions (*D*_null_). The same process was repeated with Maxent modeling based on each of the 100 pseudoreplicate datasets in each direction (i.e., by comparing either the native or invasive range with the combined range); this is typically sufficient to reject the null hypothesis with high confidence (Warren et al., 2008; Zengeya, Robertson, Booth, & Chimimba, 2013). Finally, *D*_obs_ from the native and invasive ranges were compared with *D*_null_ by a one‐tailed test. For the equivalence test, significance was determined for those values outside of the 95% confidence limits of *D*_null_. Niche divergence was inferred when *D*_obs_ < *D*_null_ (Edwards & Keogh, 2015; Warren et al., 2008).

#### 2.4.2 Reciprocal niche models

Maxent modeling was also used to generate RNMs to compare the niche shift between native and invasive ranges for the eight AIWs (Medley, 2010). For each species, a model was calibrated with the occurrences from the native range and projected onto the invasive range before being compared with the projections from model calibrated with the occurrences from the invasive range (Medley, 2010). Similarly, a model was calibrated with the occurrences from the invasive range and projected onto the native range before being compared with the projections from model calibrated with the occurrences from the native range (Medley, 2010). We used the following classes to facilitate the interpretation of niche overlap: 0–0.2 (no or very limited overlap), 0.2–0.4 (low overlap), 0.4–0.6 (moderate overlap), 0.6–0.8 (high overlap), and 0.8–1.0 (very high overlap; Rödder and Engler 2011).

## 3 Results

### 3.1 Model validation

All climatic niche models had AUC values >0.8 for both the training and test datasets (Table S1), and the training omission rates were <17% (*p *<* *.001, one‐tailed; Table S3), indicating that the models had good discriminatory power.

### 3.2 Climatic niche divergence

The climatic niches of the AIWs were significantly divergent between the native ranges (the American continent) and the invasive ranges (China), that is, *D*_obs_ < *D*_null_ for all eight AIWs using the approaches of both Broennimann et al. (2012) and Warren et al. (2008) (*p *<* *.01; Table 1 and Figure S2). *C. canadensis* had the lowest climatic niche divergence (*D*_obs_: 0.385), and *A. retroflexus* had the highest (*D*_obs_: 0.045; Table 1 and Figure S2). Based on RNM, the climatic niche overlap was moderate (*D*: 0.537 ± 0.102) in the invasive ranges and low in the native ranges (*D*: 0.376 ± 0.047), indicating moderate success in projecting to the invasive ranges and low success in projecting to the native ranges. *C. bonariensis* had the lowest climatic niche overlap in the invasive ranges (*D*: 0.351), and *P. angulata* had the lowest niche overlap in the native ranges (*D*: 0.304; Table 1).

**Table 1:** Test of the climatic niche divergence of the eight alien invasive weeds

Species	Family	*D*_obs1_	*D*_obs2_	*D*_Invasive_	*D*_Native_	Expansion	Stability
*Amaranthus retroflexus*	Amaranthaceae	**0.045**	**0.278**	0.555	0.409	0.462	0.538
*Amaranthus spinosus*	Amaranthaceae	**0.102**	**0.344**	0.624	0.337	0.235	0.765
*Amaranthus viridis*	Amaranthaceae	**0.204**	**0.273**	0.538	0.354	0.075	0.925
*Bidens pilosa*	Asteraceae	**0.214**	**0.325**	0.472	0.348	0.147	0.853
*Conyza bonariensis*	Asteraceae	**0.180**	**0.213**	0.351	0.389	0.128	0.872
*Conyza canadensis*	Asteraceae	**0.097**	**0.385**	0.719	0.468	0.300	0.700
*Galinsoga parviflora*	Asteraceae	**0.349**	**0.243**	0.478	0.395	0.036	0.964
*Physalis angulata*	Solanaceae	**0.098**	**0.248**	0.561	0.304	0.130	0.870
Mean		0.161	0.289	0.537	0.376	0.189	0.811
*SD*		0.090	0.054	0.102	0.047	0.130	0.130

*D*_obs1_ and *D*_obs2_ are the observed niche overlap (Schoener's *D*) for each alien invasive weed based on native and invasive ranges using the methods described by Broennimann et al. (2012) and Warren et al. (2008), respectively; *D*_Invasive_ and *D*_Native_ are the niche overlap in the invasive range and the native range, respectively, based on RNM; Expansion is the climatic niche expansion in the invasive range; Stability refers to the niche stability in the invasive range. Bold values represent significant climatic niche divergence (*p *<* *.05) between the native and invasive ranges.

Climatic niche expansion (within the invasive range, but outside of the native one) was 0.189 ± 0.130 in the invasive range (*A. retroflexus* with the largest expansion and lowest niche stability and *G. parviflora* with the least expansion and highest niche stability; Table 1). The average climatic niche breadth of the AIWs based on the invasive ranges, native ranges, and combined invasive and native ranges were 0.284 ± 0.046, 0.142 ± 0121, and 0.313 ± 0.077, respectively, and the ENMs differed significantly among these range classes (*t*‐test, *p *<* *.001; Table S4), indicating that the AIWs had larger climatic niche breadths in the invasive ranges than the native ranges.

### 3.3 Change in habitat suitability

**Figure 2:**
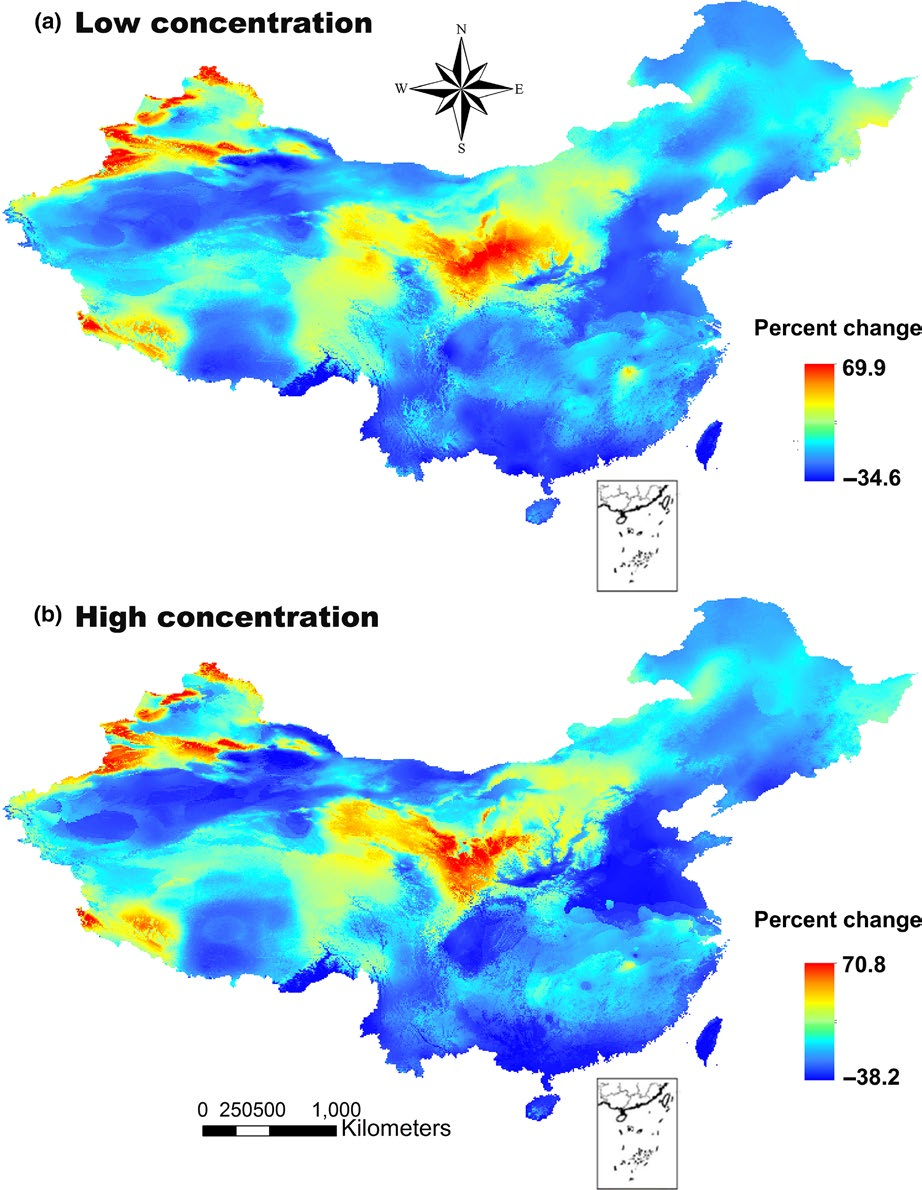
Average novel climates of all the alien invasive weeds in the low‐concentration (a) and high‐concentration (b) scenarios based on MESS maps. Values ranged from −100 to 100 percent change, with negative values indicating novel climates, and positive values indicating climates similar to the current conditions

Based on the MESS maps, novel climates for all eight AIWs were projected to occur in Heilongjiang, Jilin, Liaoning, Inner Mongolia, Gansu, Qinghai, Tibet, and Xinjiang under the low‐ and high‐concentration scenarios (Figures 2 and S1). Compared with the low‐concentration scenario, the novel climates increased under the high‐concentration scenario (Figures 2 and S1). The average habitat suitability was the highest in *C*. *canadensis* and lowest in *A. spinosus* (Table 2). The average habitat suitability of all eight AIWs across all pixels increased significantly as the gas concentration increased (0.266 ± 0.065 in the present day, 0.284 ± 0.106 under the low‐concentration scenario, and 0.296 ± 0.146 under the high‐concentration scenario; *p *<* *.05; Table 2), indicating that the high‐concentration scenario had a stronger positive effect on the habitat suitability of the AIWs than the low‐concentration scenario.

**Table 2:** Habitat suitability of the eight alien invasive weeds in the current‐, low‐, and high‐concentration scenarios and its changes between the current‐gas concentration and the low‐concentration (change—low) or the high‐concentration (change–high) scenarios

Species	Current	Low	High	Change—low (%)	Change—high (%)
*Amaranthus retroflexus*	0.336	0.434	0.501	29.1	49.1
*Amaranthus spinosus*	0.175	0.184	0.196	5.10	11.8
*Amaranthus viridis*	0.232	0.277	0.294	19.2	26.5
*Bidens pilosa*	0.253	0.170	0.105	−32.6	−58.3
*Conyza bonariensis*	0.259	0.265	0.251	2.50	−3.00
*Conyza canadensis*	0.395	0.472	0.548	19.3	38.5
*Galinsoga parviflora*	0.212	0.191	0.170	−10.2	−20.1
*Physalis angulata*	0.266	0.282	0.306	5.90	14.9
Mean	0.266	0.284	0.296	4.80	7.40
*SD*	0.065	0.106	0.146	18.2	32.3

Climate change increased the habitat suitability of AIWs in China (Table 2). The mean changes in the habitat suitability of the eight AIWs were smaller in the low‐concentration scenario than in the high‐concentration scenario (4.80 ± 18.2% in the low‐concentration scenario vs. 7.40 ± 32.3% in the high‐concentration scenario; Table 2). The change in habitat suitability was largest for *A. retroflexus* (29.1% in the low‐concentration scenario and 49.1% in the high‐concentration scenario) and smallest for *B. pilosa* (−32.6% in the low‐concentration scenario and −58.3% in the high‐concentration scenario; Table 2).

**Figure 3:**
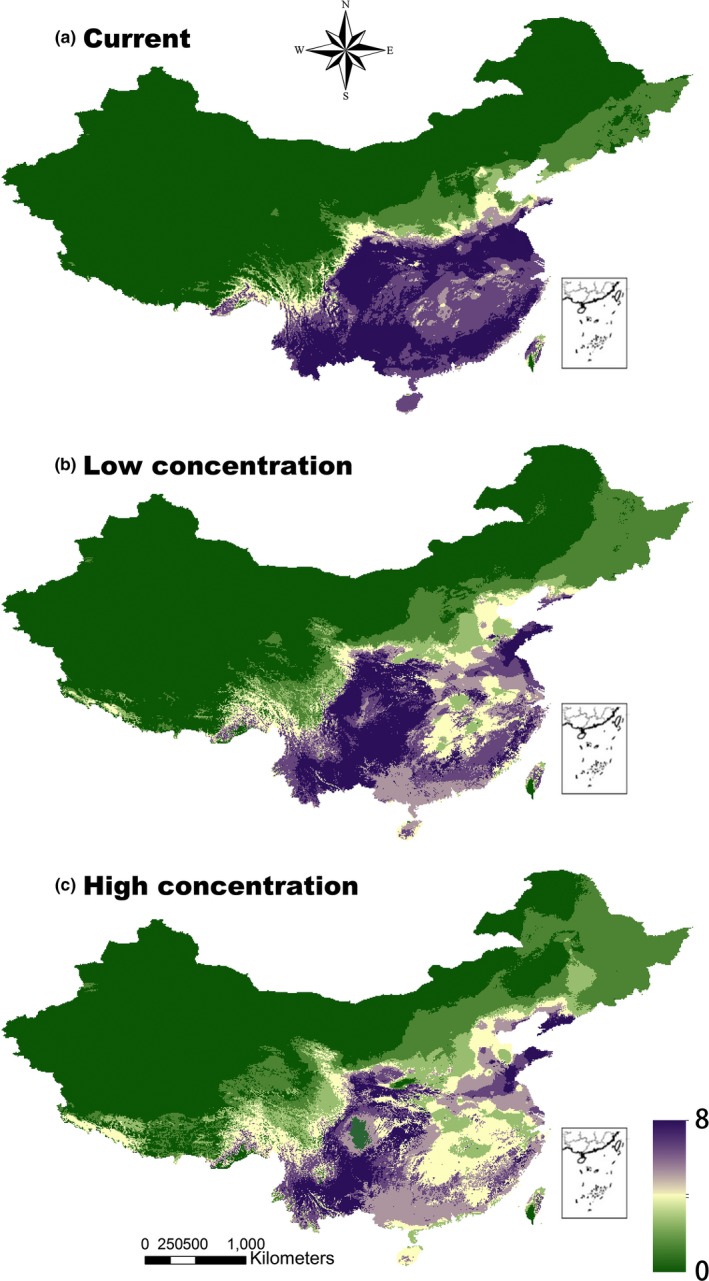
Habitat suitability of the eight alien invasive weeds in the current‐concentration (a), low‐concentration (b), and high‐concentration (c) scenarios. The minimum value for habitat suitability was 0, and the maximum value was 8

The regions with the highest habitat suitability of the AIWs were the southern provinces, including Anhui, Fujian, Guangdong, Guangxi, Guizhou, Jiangsu, Yunnan, and Zhejiang in the current‐, low‐, and high‐concentration scenarios (Table 3 and Figures 3 and S1). Furthermore, the AIWs also had high habitat suitability in Shandong under both low‐ and high‐concentration scenarios (Table 3 and Figures 3 and S1). At the province level under climate change conditions, the habitat suitability for all eight AIWs was projected to increase significantly with latitude (except under the high‐concentration scenario) and altitude but decrease significantly with longitude (*p *<* *.05; Figure S3). Compared with the present‐day scenario, the regions with the largest changes in habitat suitability included the provinces at a lower longitude (e.g., Qinghai, Tibet, and Gansu), a higher latitude (e.g., Heilongjiang, Jilin, Liaoning, Inner Mongolia, and Gansu under the low‐concentration scenario only), and a high altitude (e.g., Qinghai and Tibet; Table 3 and Figure S1). There were significant positive relationships between the change in the habitat suitability and latitude for almost all AIWs in both the low‐ and high‐concentration scenarios (*p *<* *.05; Table 4). The only exception to this trend was *G. parviflora* in the low‐concentration scenario (Table 4). Additionally, as the altitude increased, the habitat suitability of the AIWs (except for *A. retroflexus*,* C. canadensis,* and *A. viridis* in the low‐concentration scenario and *A. spinosus* in the high‐concentration scenario) were projected to increase significantly (*p *<* *.05; Table 4). The changes in habitat suitability for *C. bonariensis*,* G. parviflora*,* P. angulata,* and *C. canadensis* were significantly positively related to longitude (*p *<* *.05; Table 4).

**Table 3:** Habitat suitability of the eight alien invasive weeds in the current‐, low‐, and high‐concentration scenarios at the province scale and its changes between the current‐gas concentration and the low‐concentration (change—low) or the high‐concentration (change—high) scenarios

Province	Long. (°)	Lat (°)	Alt. (m)	Current	Low	High	Change—low (%)	Change—high (%)
Anhui	117.2	31.8	116.1	6.381	4.912	4.355	−23.0	−31.7
Fujian	118.0	26.1	476.4	6.542	5.646	4.940	−13.7	−24.5
Gansu	100.9	37.8	2067.3	1.113	1.834	2.221	64.7	99.5
Guangdong	113.4	23.3	214.3	6.540	5.379	4.534	−17.7	−30.7
Guangxi	108.8	23.8	388.2	6.745	5.618	5.064	−16.7	−24.9
Guizhou	106.9	26.8	1094.3	6.629	6.937	6.317	4.70	−4.70
Hainan	109.7	19.2	182.2	5.880	4.490	3.892	−23.6	−33.8
Hebei	116.2	39.6	501.9	1.758	2.605	3.193	48.2	81.7
Heilongjiang	127.8	47.9	312.6	0.871	1.449	1.753	66.3	101.2
Henan	113.6	33.9	239.7	4.919	4.496	4.312	−8.60	−12.3
Hubei	112.3	31.0	422.7	6.476	5.454	4.459	−15.8	−31.1
Hunan	111.7	27.6	350.2	5.903	5.220	4.536	−11.6	−23.2
Inner Mongolia	113.9	44.1	995.6	0.484	0.801	1.095	65.5	126.4
Jiangsu	119.4	33.0	12.4	6.914	5.736	5.021	−17.0	−27.4
Jiangxi	115.7	27.6	243.3	5.993	4.663	4.010	−22.2	−33.1
Jilin	126.2	43.7	403.8	1.607	1.779	2.101	10.7	30.8
Liaoning	122.6	41.3	232.1	1.920	2.704	3.865	40.8	101.3
Ningxia	106.2	37.3	1547.5	1.147	1.282	1.570	11.8	36.8
Qinghai	96.0	35.7	4029.4	0.140	0.540	1.282	286.4	818.0
Shaanxi	108.9	35.2	1118.1	3.520	4.347	3.993	23.5	13.4
Shandong	118.1	36.3	90.0	4.351	5.046	5.224	16.0	20.1
Shanxi	112.3	37.6	1162.0	1.730	2.157	2.472	24.7	42.9
Sichuan	103.5	30.5	2304.0	4.439	4.762	4.475	7.30	0.80
Taiwan	121.0	23.8	787.8	4.240	3.332	2.945	−21.4	−30.5
Tibet	88.4	31.5	4730.3	0.567	1.101	1.612	94.0	184.1
Xinjiang	85.2	41.1	1894.1	0.102	0.197	0.251	93.4	147.0
Yunnan	101.5	25.0	1878.9	6.403	6.258	5.942	−2.30	−7.20
Zhejiang	120.2	29.3	270.9	6.154	5.445	4.367	−11.5	−29.0
Mean				3.910	3.721	3.564	23.3	52.1
*SD*				2.472	1.956	1.549	61.7	159.9

**Table 4:** Relationships of the changes in habitat suitability between the current‐gas concentration and the low‐concentration (change—low) or the high‐concentration (change—high) scenario with the longitude, latitude, and altitude based on the occurrence localities of each alien invasive species

Species	Longitude		Latitude		Altitude	
	Change—low	Change—high	Change—low	Change—high	Change—low	Change—high
*Amaranthus retroflexus*	−0.163	−0.068	0.700**	0.699**	0.006	0.206*
*Amaranthus spinosus*	−0.129	−0.149	0.424**	0.386**	0.424**	0.216*
*Amaranthus viridis*	0.097	0.084	0.476**	0.466**	0.037	0.044
*Bidens pilosa*	−0.084	−0.088	0.396**	0.313**	0.231**	0.313**
*Conyza bonariensis*	−0.351**	−0.449**	0.384**	0.237*	0.549**	0.635**
*Conyza canadensis*	−0.180*	−0.156	0.576**	0.623**	0.139	0.163*
*Galinsoga parviflora*	−0.499**	−0.538**	0.199	0.225*	0.631**	0.671**
*Physalis angulata*	−0.281**	−0.335**	0.243**	0.246**	0.470**	0.612**

**p Value < .01; *p value < .05.

## 4 Discussion

We found significant climatic niche divergence for all eight AIWs between the native range (the American continent) and the invasive range (China), indicating that climatic niche divergence must be considered when using ENMs to project the habitat suitability of AIWs in invasive ranges. As suggested by previous studies, regional climatic niches may differ significantly between native and invasive ranges for invasive plant species (Dellinger et al., 2016; Early & Sax, 2014; Gallien et al., 2012). Furthermore, the values of overlap between the native and invasive niches were extremely low, and the niche expansion of some species was large, for example, for *A. retroflexus* and *C. Canadensis*. This highlights the partial niche overlap between the native and invasive ranges and the difficulty of pre‐introduction weed risk assessment (Gallien et al., 2012; Hulme, 2012; Early & Sax, 2014; Shabani & Kumar, 2015; Table 1). It is not sensible to predict the spread risk of AIWs in the invade regions based on the occurrence records of either native ranges or invasive ranges (Early & Sax, 2014). Furthermore, the low and moderate success of the AIWs in projections based on the native and invasive ranges, respectively, indicated that the AIWs are likely to expand more extensively into China than in their native ranges owing to more favorable habitat suitability in China (Collingham et al., 2000; Hoffmann & Sgrò, 2011; Warren & Seifert, 2011; Beaumont et al., 2014). Our results, together with those of previous studies (Gallien et al., 2012; Mainali et al., 2015; Shabani & Kumar, 2015), suggest that the occurrence records of both native and invasive ranges should be used in applying ENMs for the assessment of plant invasion.

The AIWs may exceed the limits of their native climatic conditions and adapt to the climatic conditions of non‐native regions (Alexander, 2013; Hoffmann & Sgrò, 2011). The climatic niche breadth of the AIWs was larger in the invasive range than in the native range, and the AIWs have wide distributions (Xu & Qiang, 2011). Furthermore, Early and Sax (2014) showed that the niche‐shift distance demonstrates the degree of climate change that species may be able to resist. In other words, AIWs could have larger climate tolerances in the invasive range than in the native range. Hence, the AIWs may be closer to equilibrium in the invasive range than in the native range (Callen & Miller, 2015; Early & Sax, 2014; Strubbe, Broennimann, Chiron, & Matthysen, 2013). Niche divergence is strongly related to rapid evolution in the invasive range, disequilibrium in the native range caused by biotic interactions, dispersal barriers, and human activities (Ansong & Pickering, 2015; Dellinger et al., 2016; Guo, Lambertini, Li, Meyerson, & Brix, 2013; Lötter & Maitre, 2014; Marini et al., 2012; Martínez‐Cabrera, Schlichting, Silander, & Jones, 2012; Schmidt & Drake, 2011). Previous studies have shown that plant species for which dispersal ability is limited in native ranges could occupy wide climatic niche spaces enabled by genetic evolution and human activities (Donoghue & Edwards, 2014; Dellinger et al., 2016). Sexual reproduction as well as self‐fertilization and asexual reproduction by clonal growth may promote climatic niche shifts during the invasion processes (Dellinger et al., 2016). Furthermore, the rapid adaptive evolution of the fundamental niches of species may result in climatic niche changes (Prentis et al. 2008; Dellinger et al., 2016). Niche divergence often involves a period of introduction by human activity, such as agriculture, transportation, and trade, with some degree of plasticity, allowing for the establishment of individual plants at least temporarily and enabling subsequent evolution (Donoghue & Edwards, 2014; González‐Moreno, Diez, Richardson, & Vilà, 2015; Guisan et al., 2014; Lötter & Maitre, 2014; Martínez‐Cabrera et al., 2012). For example, *A. retroflexus* was introduced to China as agricultural feed and expanded via agricultural transportation. Hence, to prevent and control AIW expansion in response to climatic niche divergence, it is necessary to restrict the scale of AIW colonization via human activity and prevent the escape of AIWs from agricultural areas (Lötter & Maitre, 2014; Xu & Qiang, 2011). Previous studies have shown that invasive plant species has the large potential to occupy the open niche and reach climatic equilibrium in the invasive ranges (Early & Sax, 2014; Tingley, Vallinoto, Sequeira, & Kearney, 2014; Dellinger et al., 2016). Our results provide important evidence that AIWs can exceed the climatic limits of their native climatic niches and potentially occupy a broader climatic niche of invasive ranges until niche equilibrium is reached.

We found that the eight AIWs from the American continent could potentially expand widely in southern China (i.e., into a low latitude) owing to high habitat suitability and were expected to shift to Qinghai and Tibet (regions of higher altitude) as well as Heilongjiang, Jilin, Liaoning, Inner Mongolia, and Gansu (regions of higher latitude), indicating the need for measures to prevent and control AIW invasion at the country‐wide level. In particular, this shift was more obvious in the high‐concentration scenario than in the low‐concentration scenario. These results are consistent with previous studies showing that plant species had the ability to move to some regions of higher altitude and latitude under climate change conditions (Donoghue & Edwards, 2014; Petitpierre et al., 2015; Xu & Qiang, 2011), providing a theoretical basis for the prevention and control of AIWs in China.

To address the practical issues related to the projected range extensions, we used the following measures. First, we needed to take measures to prevent and control the expansion of AIWs in southern provinces, such as Guangxi, Guizhou, Yunnan, Jiangsu, Chongqing, and Guangdong, where extensive invasion of AIWs is expected to occur under the current climatic conditions (Adhikari et al., 2015). Second, in consideration of the predicted changes in habitat suitability, we must develop an effective indicator of biological invasibility and design long‐term management plans for AIWs in Heilongjiang, Jilin, Liaoning, Inner Mongolia, Gansu, Qinghai, and Tibet, where invasion is expected to occur under climate change (Crossman, Bryan, & Cooke, 2011; Mortensen, Egan, Maxwell, Ryan, & Smith, 2012). Finally, we need to attach importance to the prevention and control of *A. retroflexus* expansion because this species has very high habitat suitability and could have clear increases in habitat suitability in the future (Beaumont et al., 2014; Costa et al., 2013; Sheppard, 2013).

Some studies have shown that climatic niche conservatism exists widely among terrestrial plant invaders, such that the transferability of ENMs supports the projection of habitat suitability of plant species in the invasive ranges based on observed habitat suitability in the native ranges (Petitpierre et al., 2012, 2015; Callen & Miller, 2015). However, many factors, including human activity and climate change, may promote the adaptation of plant species to novel climatic conditions in non‐native ranges and result in climatic niche divergence between native and invasive ranges (González‐Moreno et al., 2015; Martínez‐Cabrera et al., 2012). Our findings provide strong support for this argument and suggest that climatic niche shifts must be evaluated before examining the potential distribution of plant invaders using ENMs, especially under climate change conditions (Warren & Seifert, 2011).

In conclusion, our findings provide a solid basis for the use of ENMs and raise questions about the mechanistic underpinnings of broadscale geographic patterns. We analyzed the impact of nonclimatic factors changes across time and space on the niche shift of AIWs (and even invasive plant species) between native and invasive ranges and measured the potential effects of AIWs on the economy and ecosystem of the invasive regions in the future due to climate change and integrated ENMs into the management of invasion risks.

## Conflict of Interest

None declared.
