# Supplementary material for: Climatic niche divergence and habitat suitability of eight alien invasive weeds in China under climate change
Source: Ecol Evol. 2017 Feb 8;7(5):1541–52. doi: 10.1002/ece3.2684 (PMC5330889; doi:10.1002/ece3.2684)

**Supporting materials**

**Table S1.** Summary of the alien invasive weeds

| Species | Family | All | | |  | Invasive | |  |  | Native | | |
| --- | --- | --- | --- | --- | --- | --- | --- | --- | --- | --- | --- | --- |
|  |  | Record | AUC_training_ | AUC_test_ |  | Record | AUC_training_ | AUC_test_ |  | Record | AUC_training_ | AUC_test_ |
| *Amaranthus retroflexus* | Amaranthaceae | 204 | 0.956 | 0.944 |  | 95 | 0.983 | 0.978 |  | 109 | 0.965 | 0.952 |
| *Amaranthus spinosus* | Amaranthaceae | 528 | 0.939 | 0.933 |  | 90 | 0.991 | 0.990 |  | 438 | 0.951 | 0.945 |
| *Amaranthus viridis* | Amaranthaceae | 183 | 0.952 | 0.938 |  | 73 | 0.991 | 0.989 |  | 110 | 0.955 | 0.937 |
| *Bidens pilosa* | Asteraceae | 1004 | 0.921 | 0.916 |  | 128 | 0.990 | 0.987 |  | 876 | 0.935 | 0.931 |
| *Conyza bonariensis* | Asteraceae | 382 | 0.938 | 0.933 |  | 83 | 0.991 | 0.990 |  | 299 | 0.950 | 0.943 |
| *Conyza canadensis* | Asteraceae | 435 | 0.925 | 0.914 |  | 154 | 0.984 | 0.979 |  | 281 | 0.936 | 0.922 |
| *Galinsoga parviflora* | Asteraceae | 186 | 0.970 | 0.962 |  | 82 | 0.992 | 0.991 |  | 104 | 0.967 | 0.960 |
| *Physalis angulata* | Solanaceae | 476 | 0.924 | 0.914 |  | 140 | 0.987 | 0.985 |  | 336 | 0.939 | 0.929 |
| Mean |  | 429 | 0.941 | 0.932 |  | 106 | 0.989 | 0.986 |  | 319 | 0.950 | 0.940 |
| SD |  | 256 | 0.016 | 0.016 |  | 29 | 0.003 | 0.005 |  | 240 | 0.012 | 0.012 |

All, Invasive, and Native represent the climatic niche breadth of each alien invasive weed from its combined native and invasive range, invasive range only, or native range only, respectively. The record represents the number of occurrence localities used as the input for Maxent modeling.

**Table S2.** Summary of bioclimatic variables that were used as bioclimatic layers to model the habitat suitability of alien invasive weeds with Maxent.

| Code | Environmental variables | Unit |
| --- | --- | --- |
| Bio1 | Annual mean temperature | °C |
| Bio2 | Mean diurnal range | °C |
| Bio4 | Temperature seasonality | SD*100 |
| Bio8 | Mean temperature of the wettest quarter | °C |
| Bio12 | Annual precipitation | mm |
| Bio14 | Precipitation of the driest month | mm |
| Bio15 | Precipitation seasonality | CV |
| Bio18 | Precipitation of the warmest quarter | mm |

SD represents the standard deviation; CV represents the coefficient of variation.

**Table S3.** The training omission rate of alien invasive weeds.

| Name | All | Invasive | Native |
| --- | --- | --- | --- |
| *Amaranthus retroflexus* | 0.052±0.041 | 0.021±0.030 | 0.059±0.023 |
| *Amaranthus spinosus* | 0.042±0.043 | 0.031±0.028 | 0.057±0.025 |
| *Amaranthus viridis* | 0.035±0.042 | 0.054±0.023 | 0.031±0.029 |
| *Bidens pilosa* | 0.048±0.048 | 0.035±0.030 | 0.038±0.038 |
| *Conyza bonariensis* | 0.064±0.048 | 0.021±0.032 | 0.030±0.033 |
| *Conyza canadensis* | 0.067±0.047 | 0.027±0.029 | 0.038±0.033 |
| *Galinsoga parviflora* | 0.041±0.035 | 0.030±0.024 | 0.021±0.029 |
| *Physalis angulata* | 0.045±0.051 | 0.029±0.029 | 0.054±0.025 |

All, Invasive, and Native represent the training omission rate of the Maxent modeling from the combined native and invasive range, from the invasive range only, and from the native range only, respectively. For each alien invasive weed, the values presented are the mean ± SD across nine common thresholds (Phillips et al. 2006). The nine common thresholds included (1, 2, and 3) fixed cumulative values of 1, 5, and 10, respectively, (4) a minimum training presence, (5) the tenth percentile training presence, (6 and 7) equal training and maximum training plus specificity, respectively, (8) balanced training omission, a predicted area, and a threshold value, and (9) equal entropy of the thresholded and original distributions (Phillips et al. 2006).

**Table S4.** The climatic niche breadth of the alien invasive weeds.

| Name | All | Invasive | Native |
| --- | --- | --- | --- |
| *Amaranthus retroflexus* | 0.456 | 0.360 | 0.430 |
| *Amaranthus spinosus* | 0.215 | 0.225 | 0.037 |
| *Amaranthus viridis* | 0.249 | 0.246 | 0.107 |
| *Bidens pilosa* | 0.297 | 0.278 | 0.082 |
| *Conyza bonariensis* | 0.302 | 0.295 | 0.093 |
| *Conyza canadensis* | 0.416 | 0.350 | 0.226 |
| *Galinsoga parviflora* | 0.275 | 0.245 | 0.103 |
| *Physalis angulata* | 0.294 | 0.277 | 0.057 |
| Mean ± SD | 0.313 ± 0.077 | 0.284 ± 0.046 | 0.142 ± 0.121 |

All, Invasive, and Native represent the climatic niche breadth for each alien invasive weed from its combined native and invasive range, invasive range only, and native range only, respectively.

**Fig. S1.** The study area

**
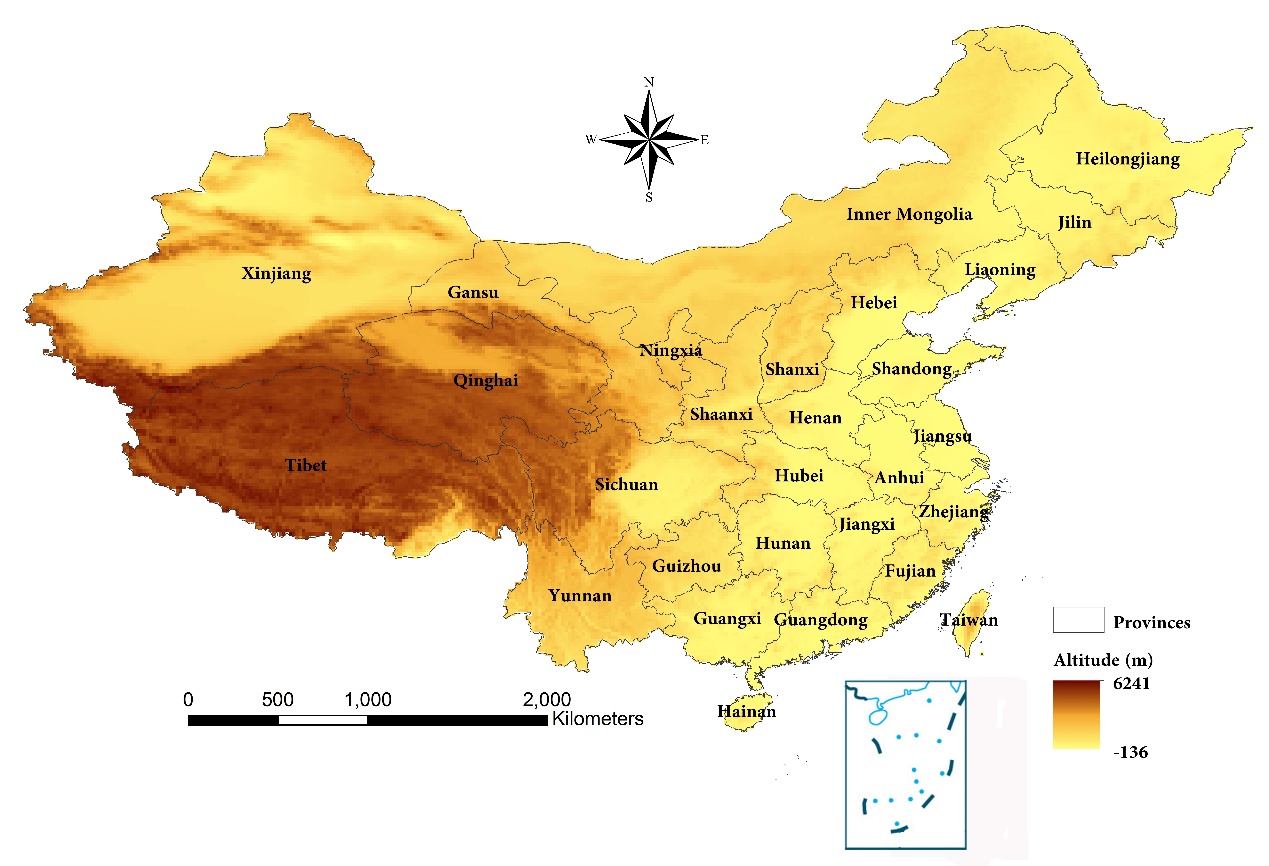
**

**Fig. S2.** Frequency histograms from tests of niche divergence for eight AIWs using the method described by Warren et al. (2008). The red bar represents the frequency for 100 niche overlap scores (namely, Schoener’s D) generated under the null distributions for each AIW. Observed niche similarity values (*D*_obs_; arrows) were compared to the null distributions for each species pair. *D*_obs_ values that are lower than the null distribution support niche divergence.


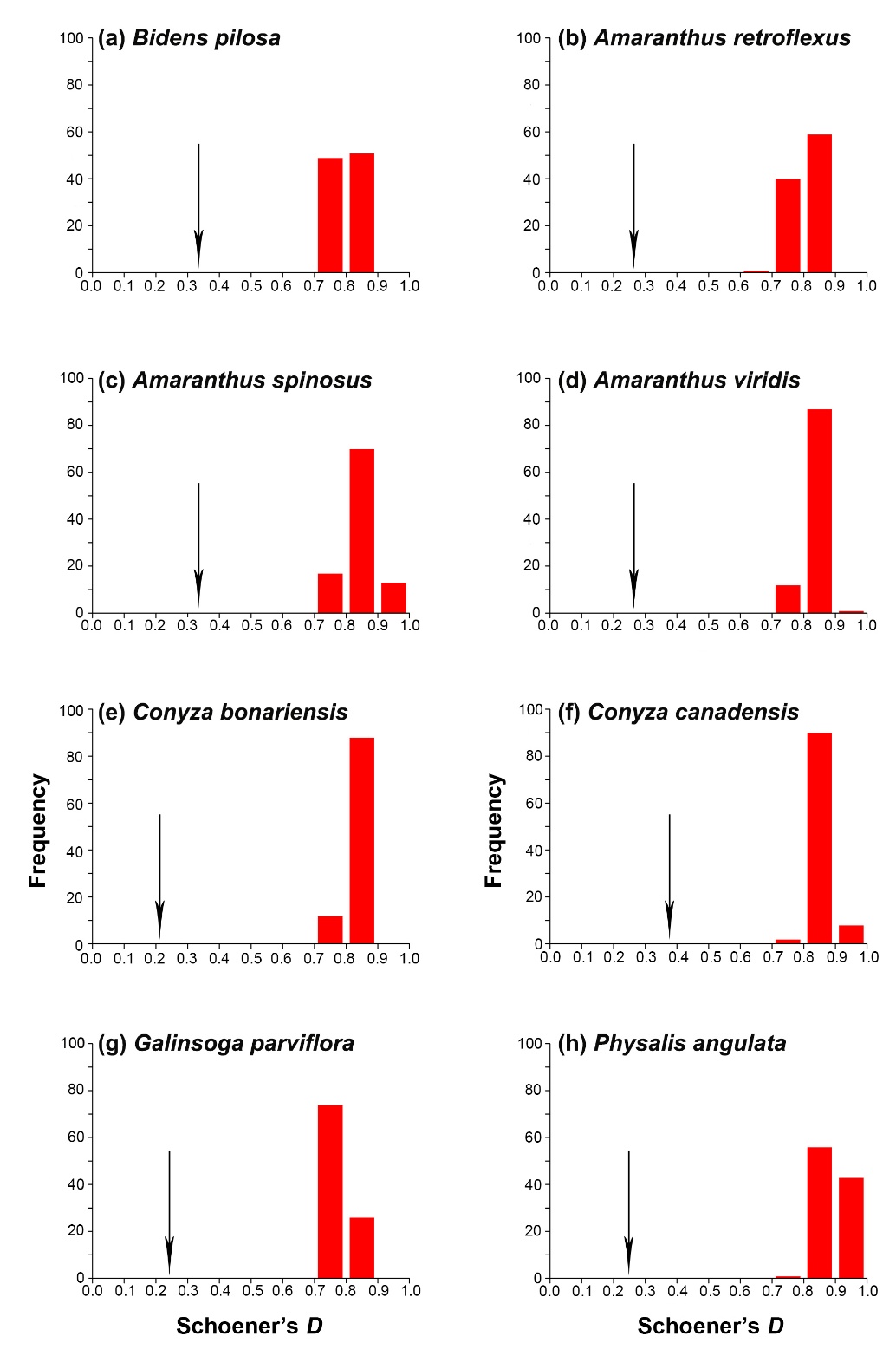


**Fig. S3.** Relationships between longitude, latitude, and altitude, respectively, and the change in habitat suitability between the present-day scenario and the low (a, c, and e) or the high (b, d, and f) concentration scenarios at the province scale. Change-Low and Change-High represent changes in habitat suitability between the present-day gas concentration and the low and high concentration scenarios, respectively.
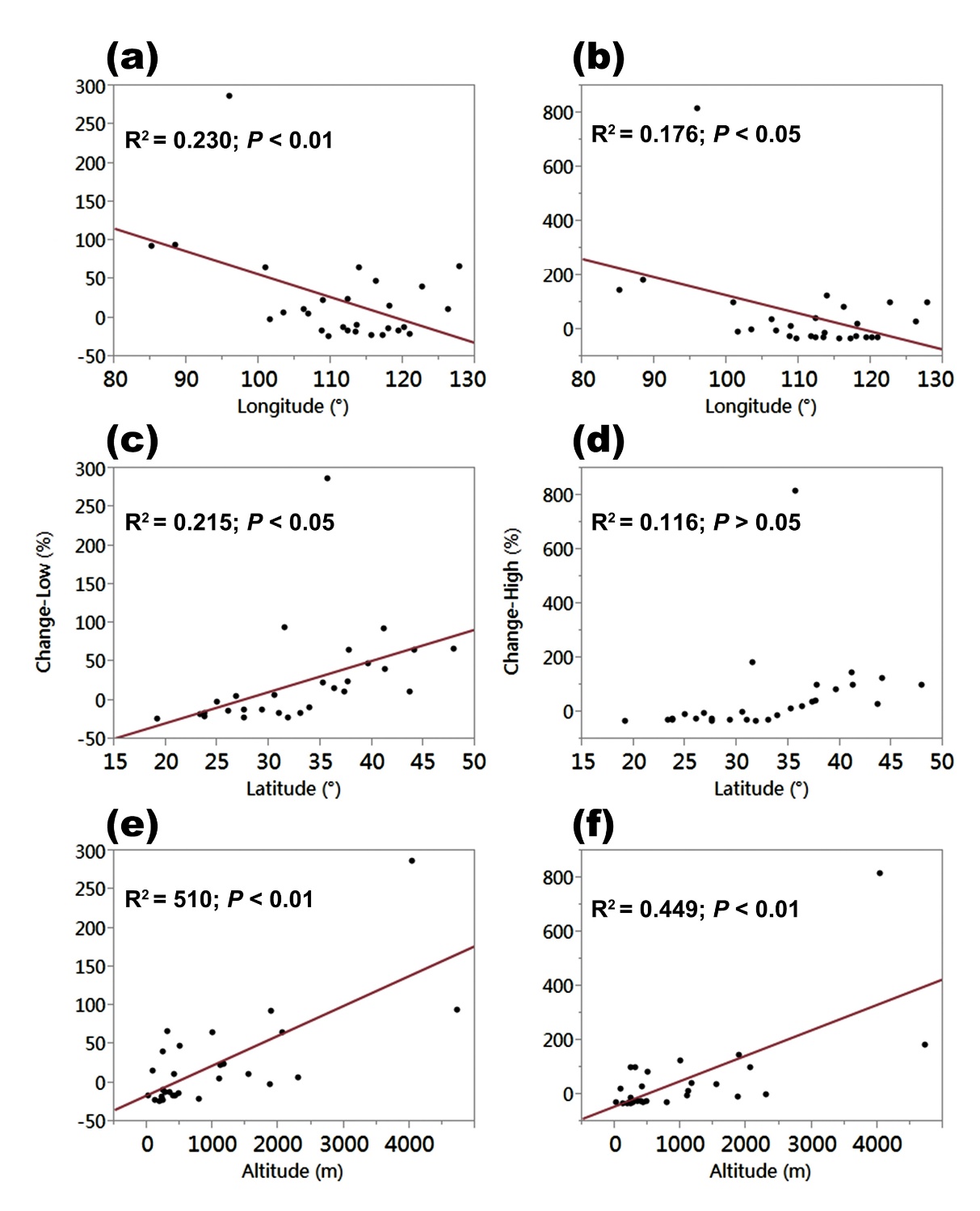

Supplement: Supplementary file 1 [file ECE3-7-1541-s001.docx]
